# Examination of Genome Homogeneity in Prokaryotes Using Genomic Signatures

**DOI:** 10.1371/journal.pone.0008113

**Published:** 2009-12-02

**Authors:** Jon Bohlin, Eystein Skjerve

**Affiliations:** Department of Food Safety and Infection Biology, Norwegian School of Veterinary Science, Oslo, Norway; University of Stellenbosch, South Africa

## Abstract

**Background:**

DNA word frequencies, normalized for genomic AT content, are remarkably stable within prokaryotic genomes and are therefore said to reflect a “genomic signature.” The genomic signatures can be used to phylogenetically classify organisms from arbitrary sampled DNA. Genomic signatures can also be used to search for horizontally transferred DNA or DNA regions subjected to special selection forces. Thus, the stability of the genomic signature can be used as a measure of genomic homogeneity. The factors associated with the stability of the genomic signatures are not known, and this motivated us to investigate further. We analyzed the intra-genomic variance of genomic signatures based on AT content normalization (0^th^ order Markov model) as well as genomic signatures normalized by smaller DNA words (1^st^ and 2^nd^ order Markov models) for 636 sequenced prokaryotic genomes. Regression models were fitted, with intra-genomic signature variance as the response variable, to a set of factors representing genomic properties such as genomic AT content, genome size, habitat, phylum, oxygen requirement, optimal growth temperature and oligonucleotide usage variance (OUV, a measure of oligonucleotide usage bias), measured as the variance between genomic tetranucleotide frequencies and Markov chain approximated tetranucleotide frequencies, as predictors.

**Principal Findings:**

Regression analysis revealed that OUV was the most important factor (*p<0.001*) determining intra-genomic homogeneity as measured using genomic signatures. This means that the less random the oligonucleotide usage is in the sense of higher OUV, the more homogeneous the genome is in terms of the genomic signature. The other factors influencing variance in the genomic signature (*p<0.001*) were genomic AT content, phylum and oxygen requirement.

**Conclusions:**

Genomic homogeneity in prokaryotes is intimately linked to genomic GC content, oligonucleotide usage bias (OUV) and aerobiosis, while oligonucleotide usage bias (OUV) is associated with genomic GC content, aerobiosis and habitat.

## Introduction

Analyses of the DNA composition in prokaryotes and eukaryotes have revealed important differences. While prokaryotes have, on average, a higher fraction of coding DNA than eukaryotes, the latter has a seemingly more advanced DNA composition with large, non-protein coding regions [1]. In addition, the DNA molecule in eukaryotic organisms is larger and nucleosomes are used to compact it introducing pronounced, small scale (sequences consisting of approximately 200 bp), long-range correlation effects not present in bacteria [2]. In bacteria however, small scale genomic DNA (*i.e.* genetic sections covering 200 bp) has a Brownian motion, or random walk reminiscent composition, in other words, the long-range correlation effects described above for eukaryotes are absent in microbial genomes [3]. The random walk-like base composition pattern found in prokaryotic genomes [1] indicates that statistical methods based on random walk methodology, also known as Markov chains, may be a useful tool to model and understand prokaryotic genome composition.

Markov chains describe a set of stochastic processes that all share the Markov property. This property states, in common terms, that the probability that an event occurs in the future is only dependent on the present and independent of any other events. In other words, Markov chains are, in general, only concerned with what happens in the last time step and not the previous history to predict a future event, hence the term “random walk” [4]. Markov chains can be extended to be made dependent on additional events, or time steps, allowing for short range correlation effects, *i.e.* short term memory, in the random walk process [4]. Short range correlated Markov chains are known as *n*'th order Markov chains, where *n* denotes the number of dependent time-steps, or events [4].

Markov chain theory has found many applications in biology and bioinformatics and are widely used in gene-finding [5], DNA sequence search [6], rRNA gene localization [7], and protein structure identification [8]. In this study, we used Markov chains to analyze prokaryotic genome composition. This was carried out by studying the genomic frequencies of small tuples of nucleotides known as oligonucleotides. Examples of genomic oligonucleotide frequencies include nearest neighbor frequencies (dinucleotide frequencies), codon frequencies (trinucleotides) and tuples of four nucleotides, known as tetranucleotide frequencies. Dinucleotide frequencies are associated with DNA structural features and base stacking energies [9]. Codons code for amino acids in all living organisms. Since there are 64 different codon combinations, but only 20 different amino acids, multiple codons can code for the same amino acid. Closely related species often prefer the same codons for specific amino acids [10]. There are however indications that codon preference is just as much driven by environmental factors as phylogeny [11]–[13]. Tetranucleotide patterns are influenced by biases from mono- to trinucleotide frequencies [14]. Moreover, tetranucleotide patterns with corresponding structural features are similarly distributed throughout prokaryotic genomes [15], and have also been found to carry a taxonomic signal [15]–[17]. As discussed above, prokaryotic DNA has been found to follow a short range correlated, random walk like pattern that can be modeled using Markov chain analysis.

To test the genomic sequences for random walk properties, or lack thereof, we computed the variance difference between genomic oligonucleotide frequencies and Markov chain approximated oligonucleotide frequencies. Lower variance between genomic oligonucleotide frequencies and Markov chain approximated oligonucleotides implies more random walk like properties. Due to the features described above for tetranucleotide frequencies, Markov chain analysis was used to approximate genomic tetranuclenucleotide frequencies with the genomic frequencies of smaller DNA words (*i.e.* mono- to trinucleotide frequencies). Higher variance (squared difference) between genomic and approximated tetranucleotide frequencies is correlated with bias. Hence, stronger bias is in the present study taken to mean that the variance between genomic tetranucleotide frequencies and the Markov chain based random walk models is high. The more biased a genome is said to be, the more difficult it is to approximate the genomic tetranucleotide frequencies using random walk based methods such as Markov chains.

A zero'th order Markov chain (ZOM) approximates genomic oligonucleotide frequencies using the corresponding genomic nucleotide frequencies (see materials and methods for more details). For the ZOM-based approximation scheme, we assume that the lower variance between genomic and approximated tetranucleotide frequencies, the more mutated, or randomly composed, a genome is. Since each oligonucleotide frequency is approximated by the oligonucleotide's corresponding nucleotide frequencies, the ZOM approximation assumes that each nucleotide, in the oligonucleotide that is being approximated, is independent of its neighbors.

Nearest-neighbor effects, or short range correlations, are important factors in both genomic DNA structure and DNA sequence and such effects are largely responsible for the bias in the ZOM variance model discussed above. For instance, nearest neighbor nucleotides are associated with base stacking energies [9], DNA helix structure [9] and DNA structure in general [18], [19]. The three nucleotides in each codon are also dependent on each other, and this dependency is largely responsible for the preference of some codons over others that code for specific amino acids [10]. The dependencies between the nucleotides in each codon is thus strongly linked to codon usage bias in prokaryotic genomes [10]. Thus, it is clear that short range dependencies play an important role in genomic DNA composition.

Dependence of nearest neighbor nucleotides in a random walk model can be modeled using a first or second order Markov chain. A first order Markov chain (FOM) approximates genomic oligonucleotide frequencies using the oligonucleotide's corresponding mono- and dinucleotide frequencies. Hence, weak dependencies are incorporated into the FOM model by the use of genomic mono- and dinucleotide frequencies to approximate the frequencies of larger oligonucleotides as compared to only mononucleotide frequencies in the ZOM model. Even stronger neighboring effects, or short range correlations, are incorporated into the second order Markov chain (SOM), which uses di- and trinucleotide frequencies to approximate larger oligonucleotides.

**Figure 1:**
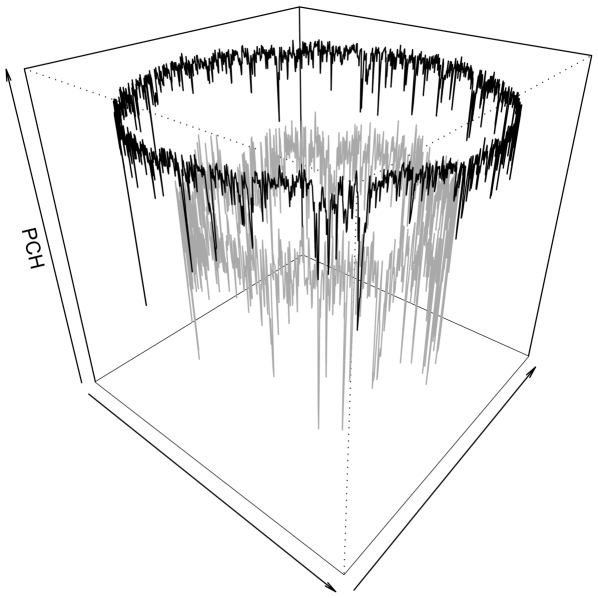
Oligonucleotide usage variance (OUV) in *Bacillus cereus* and *Pirelulla* sp. The figure shows how tetranucleotide usage varies within the *Bacillus cereus* ATCC 14579 (grey line) and *Rhodopirellula baltica* SH 1 (black line) chromosomes. The vertical axis (OUV) is a measure of oligonucleotide usage variance. Higher OUV values indicate more biased tetranucleotide usage as compared to a randomly constructed DNA sequence with corresponding AT content. It can be seen that the *R. baltica* genome has, on average, more biased tetranucleotide usage than the *B. cereus* genome.

The lower the variance is between genomic tetranucleotide frequencies and FOM and SOM based tetranucleotide frequency approximations, the stronger are the interactions of two and three neighboring nucleotides in the respective models. The variance tests measuring the random walk like behavior of the genomic DNA sequences are referred to as oligonucleotide usage variance (OUV) [14], [15]. Hence, OUV is here a measure of tetranucleotide usage bias, measured as the variance between genomic tetranucleotide frequencies and Markov-chain approximated tetranucleotide frequencies. The higher the OUV value, the more biased (*i.e.* less random walk like) we say a genome is. Conversely, smaller OUV values are taken to mean that a genome has a more random walk or Brownian motion like sequence structure corresponding to the Markov model used. In other words, while FOM and SOM models emphasize dependence between 2 and 3 nucleotides in a DNA sequence, the ZOM model assumes no such dependencies at all. ZOM based approximations are thus assumed to better model random mutations in DNA sequences, while FOM and SOM based approximations are more suited to model neighboring dependencies and short range correlations, respectively. Figure 1 shows how OUV varies in two bacterial genomes, *Bacillus cereus* ATCC 14579 and *Rhodopirellula baltica* SH 1.

The odds-ratio of genomic oligonucleotide frequencies divided by Markov chain approximated oligonucleotide frequencies, on arbitrary bulks of 50 kbp, has been shown to correspond remarkably well with known phylogenies for closely related organisms [20], [21]. The discovered phylogenetic signal made Karlin and co-authors dub the odds-ratio of observed oligonucleotide frequencies divided by approximated oligonucleotide frequencies as “genomic signatures” [22]. The stable property of the odds-ratio between observed oligonucleotide frequencies and Markov chain approximated oligonucleotide frequencies in genomic DNA, was first discovered using a dinucleotide based zero'th order Markov chain [23]. Although this finding dates back to early 1960's, it was Karlin and co-workers who discovered the more general validity of the method and called it a “genomic signature” [22]. Karlin and co-workers also tested an odds-ratio model based on a second order Markov chain model, but could not detect any improvement in performance compared to the ZOM-based odds-ratio model [20]. Subsequent studies have given a mixed picture regarding the genomic signature obtained with a SOM-based odds-ratio model compared to ZOM-based genomic signatures [16], [24], [25]. However, ZOM-, FOM- and SOM-based odds-ratios reflect taxonomical signals in prokaryotic genomes. The FOM-based odds-ratio model is especially suited to model nearest neighbor interactions between nucleotides, and may therefore be somewhat more biased towards base stacking energies than the ZOM model. Table 1 gives an overview of the different Markov chain models used in the present study together with the corresponding assumptions and biases.

**Table 1:** Assumptions of the Markov chain models and the corresponding reflected bias.

Approximation model	Oligonucleotide(s) used in approximation	Assumptions	Bias
ZOM	mononucleotide frequencies	no correlations between neighboring nucleotides	random mutations
FOM	mono- and dinucleotide frequencies	correlations between neighboring nucleotides	base stacking energies
SOM	di- and trinucleotide frequencies	Correlations between all adjacent nucleotides	base stacking energies, DNA structure, codon bias

The table shows the different assumptions and biases associated with the corresponding Markov chain model used to approximate genomic oligonucleotide frequencies.

**Figure 2:**
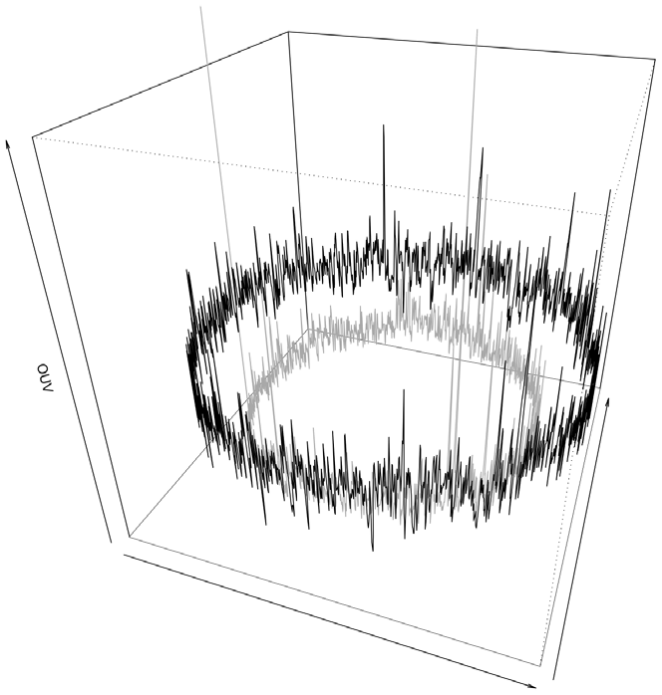
Genomics signature variance in *Bacillus cereus* and *Pirelulla sp*. The figure shows how the genomic signature varies within one of the most homogeneous chromosomes, *Rhodopirellula baltica* SH 1 (black line), and within one of the most heterogeneous chromosomes, *Bacillus cereus* ATCC 14579 (grey line). The vertical axis representing PCH, gives a measure of how homogeneous a genome is. The higher the PCH value, the more homogeneous the chromosome. It can be seen that PCH is both higher and with less variation in the *R. baltica* genome as compared to the *B. cereus* genome. While *R. baltica* is a slow growing GC rich bacterium with a relatively large genome (7 mbp), *B. cereus* is a fast growing AT rich bacterium with a genome of approximately 5.5 mbp.

Genomic signature variances within genomes can be measured using odds-ratios of genomic oligonucleotide frequencies divided by approximated oligonucleotide frequencies from smaller chunks of DNA, ranging from a few to a hundred kbps, and compared to the corresponding odds-ratios for the whole DNA sequence [25]. The genomic signature varies little within prokaryotic genomes [21], [25]. However, variations of the genomic signature may be indicative of foreign DNA from plasmids, virus or the environment being integrated into a genome [26]. Variations in genomic signatures within prokaryotic genomes is therefore occasionally linked to virulence and pathogenicity islands [14], [21], [26]. By using the Pearson correlation coefficient (*r*), giving the value 1 for complete correlation and the value 0 for no correlation, as a measure for comparing DNA sequences, it was observed [25] that considerably smaller bulks of DNA could be used to search for foreign DNA than the 50 kbp bulks of DNA first proposed [20]. The ability to detect genomic signature difference with less DNA facilitates the identification of smaller regions of DNA that may be associated with pathogenesis [14]. Analysis of dinucleotide-based genomic signature variance within *Thermotoga maritima* revealed that correlation scores as high as *r>0.9* could be obtained between genomic signatures from 5 kbp sliding windows and whole chromosome based signatures [14]. Indeed, for the same genome and sliding window size tetranucleotide-based genomic signatures obtained correlation scores of *r>0.8* [14]. In the *Bacillus subtilis* genome the average correlation score was somewhat lower than the score obtained for *T. maritima* using tetranucleotide-based genomic signatures. Although both organisms are known to have acquired considerable amounts of foreign DNA [27], [28], the average variance of the genomic signature within each genome varied considerably between the two genomes [14]. We shall refer to average variation measures of genomic signatures based on Pearson correlation as Pearson correlation-coefficient homogeneity tests (PCH). Figure 2 shows how the genomic signature, as measured using the PCH measure, varies within two genomes, *Rhodopirellula baltica* SH 1 and *Bacillus cereus* ATCC 14579.

The difference in average genomic signature variance between the bacteria discussed above motivated us to investigate genomic homogeneity in sequenced prokaryotic genomes by utilizing the stable property reflected by the Markov chain based genomic signature methods. The aim was to explore how genomic homogeneity, as measured by tetranucleotide-based genomic signatures, varied within all sequenced prokaryotic genomes, and whether this variance could be attributed to specific phylogenetic and environmental factors. Moreover, we wanted to examine the DNA compositional random walk like properties in each sequenced prokaryotic genome, and whether it could be linked to genomic homogeneity (PCH), and if it could be attributed to specific phylogenetic and environmental factors.

To model the factors affecting genomic homogeneity in prokaryotes, a linear regression analysis was used with PCH as the response variable with the predictor variables: growth temperature (a categorical factor classifying organisms as psychrophilic, mesophilic or thermophilic), AT content, chromosome size, habitat (a categorical factor describing the organisms habitat as aquatic, host-associated, multiple, specialized or terrestrial) and phyla, in addition to the corresponding Markov chain OUV.

To examine factors influencing the random walk like behavior of genomic DNA sequences, a linear regression model was set up with ZOM, FOM and SOM OUV as response variables to the following predictor variables: growth temperature, AT content, chromosome size, habitat and phyla.

Separate models were fitted for whole chromosomes, including coding and non-coding regions, and open reading frames (orfs) to measure whether any differences in the PCH and OUV measures could be detected between coding and non-coding regions.

## Results

### OUV Regression Models

**Figure 3:**
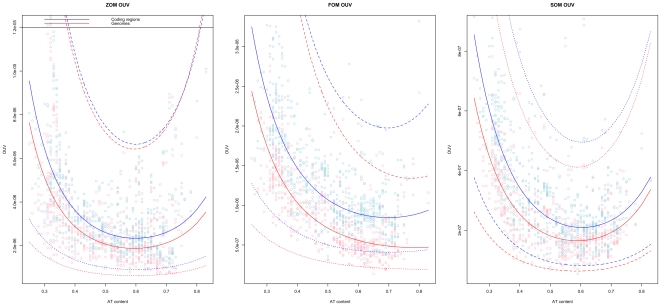
Oligonucleotide usage variance (OUV) based on ZOM, FOM and SOM models. OUV scores based on ZOM (left), FOM (middle), and SOM (right) measures are found on the vertical axis, with each respective chromosome, sorted from left to right by increasing AT content, on the horizontal axis. Red lines indicate whole chromosome OUV scores, including both coding and non-coding section, while blue lines represent concatenated open reading frames. Lower values mean better OUV approximations. Dotted lines represent 99% prediction intervals.

In Table 2 it can be seen that AT content and phyla were the strongest contributing factors in the OUV-based regression models. This means that the random walk like properties of genomic DNA in prokaryotes is, first and foremost, associated with genomic AT content (Figure 3) and phylogeny. The higher the genomic AT content, the more random walk like the genomic DNA sequence pattern tend to be. Oxygen requirement was associated with genomic base composition as measured by the OUV measure (*p<0.001*) for both FOM and SOM models. The results from the regression model indicate that aerobic organisms have a more biased genome compared to the FOM and SOM based random walk models. Habitat was associated with OUV for all models but the FOM model (*p<0.001*), meaning that the random walk like sequence structure in prokaryotic DNA is also affected by environmental conditions. Growth temperature was associated with FOM and SOM OUV (*p<0.001*), but only slightly in terms of AIC and *R^2^* scores. Hence, it is likely that growth temperature has an effect on genomic DNA composition, but that it is one of many factors involved. Chromosome size was only found to be associated with FOM and SOM orfs models (*p<0.001*), it is therefore unclear how direct the impact of genome size is on DNA composition in prokaryotes. It is known that AT content is strongly associated with genome size [14], [29], and it is therefore possible that the link observed between the FOM and SOM orfs models and genome size is a confounding factor. Table 2 shows that the coefficient of determination (*R^2^*) increased for all OUV-based regression models when restricted to open reading frames (orfs). This means that the statistical models were better at explaining variance in open reading frames than in genomic DNA sequences containing both coding and non-coding DNA.

**Table 2:** OUV regression AIC/Coefficient of variation scores.

	Constant	Size	AT	Phyla	Oxygen	Habitat	Growth temperature	Transform
ZOM	1187		909, R^2^ = 0.33	649, R^2^ = 0.55		646, R^2^ = 0.55		log
ZOM orfs	1056		683, R^2^ = 0.42	402, R^2^ = 0.62	397, R^2^ = 0.62	390, R^2^ = 0.63		log
FOM	−4399	−4463, R^2^ = 0.09	−5515, R^2^ = 0.54	−5695, R^2^ = 0.65	−5715, R^2^ = 0.66		−5717, R^2^ = 0.67	log
FOM orfs	−4204		−4757, R^2^ = 0.55	−4954, R^2^ = 0.67	−4961, R^2^ = 0.67	−4967, R^2^ = 0.68		log
SOM	961		542, R^2^ = 0.45	324, R^2^ = 0.61	314, R^2^ = 0.62	308, R^2^ = 0.62	295, R^2^ = 0.63	log
SOM orfs	2544	2511. R^2^ = 0.05	2033, R^2^ = 0.52	1766, R^2^ = 0.68	1763. R^2^ = 0.68	1757, R^2^ = 0.69		log

Results of forward fitting regression models with the response variable in the leftmost column followed by the included predictors in the subsequent columns.

From Figure 3 it can be seen that OUV scores were noticeably higher in open reading frames for all models when compared to AT content. Thus, open reading frames have a less random walk like sequence structure than non-coding regions.

**Figure 4:**
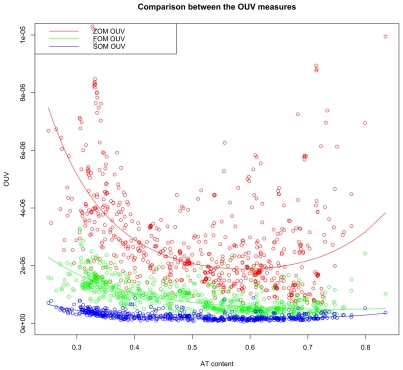
Overview of Markov model based oligonucleotide approximations in prokaryotes. OUV scores based on 0^th^, 1^st^ and 2^nd^ order Markov models (ZOM, FOM, and SOM respectively) are found on the vertical axis. Each chromosome is sorted with respect to increasing AT content from left to right along the horizontal axis. ZOMs (red line) approximate genomic tetranucleotide usage with nucleotide frequencies, while FOMs (green line) use genomic dinucleotide content in addition. The 2^nd^ order Markov model (blue line) bases tetranucleotide frequency approximations on genomic di- and trinucleotide usage. Larger OUV values mean poorer approximations which is a consequence of more biased tetranucleotide usage.

OUV scores dropped when the order of the Markov model increased (Figure 4), indicating dependence and strong interactions between neighboring nucleotides in all sequenced prokaryotic genomes examined.

From Table 2 it can be seen that the ZOM-based regression model explained the least observed variance (*R^2^ = 0.55*), while the SOM model restricted to open reading frames explained the most variance (*R^2^* = *0.69*).

ZOM OUV compared to FOM OUV scores obtained *R^2^ = 0.39*. ZOM OUV compared to SOM OUV scores were the least associated of all measures with *R^2^ = 0.3*, while FOM OUV compared to SOM OUV scores obtained the highest coefficient of determination of *R^2^ = 0.57*. In summary, this indicates that the ZOM OUV model resembled the FOM OUV model more than the SOM OUV model.

### PCH Regression Models

**Figure 5:**
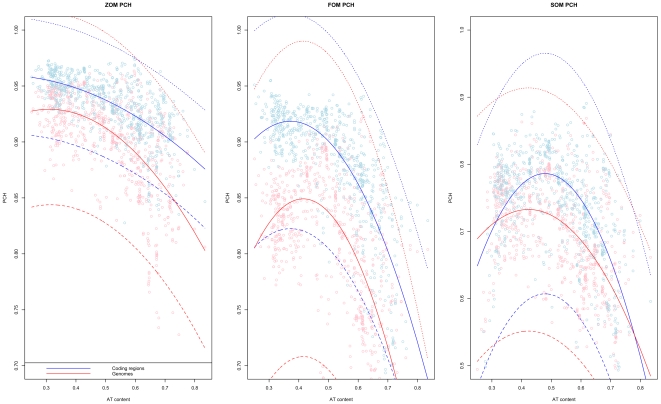
Markov chain model based PCH scores in prokaryotes. ZOM (left), FOM (middle) and SOM (right) PCH values (vertical axis) obtained for each chromosome sorted from left to right by increasing AT content (horizontal axis). The PCH scores show how the Markov chain based genomic signatures change, on average, within each chromosome. For all models we find that PCH scores are noticeably higher in coding regions (blue lines) than chromosomes, containing both coding and non-coding regions (red lines). Higher PCH values mean more homogeneous chromosomes while lower PCH means more heterogeneous chromosomes with respect to the corresponding Markov-chain based genomic signatures. Dotted lines represent 99% prediction intervals.

From Table 3 it can be seen that all Markov model based PCH regression models were influenced by AT content, respective order Markov model based OUV scores, and phyla. Thus, genomic DNA homogeneity as measured by the intra-genomic variance of Markov chain based genomic signatures increased with GC content and OUV. The more biased, *i.e.* less random walk like, the genomic DNA compositions was, the more homogeneous the genomic DNA sequence in terms of the Markov chain based genomic signature was found to be. Oxygen requirement was associated with increased genome homogeneity in all regression models except the ZOM model (*p<0.001*), while chromosome size was only found to be significant for the FOM orfs model. As was mentioned above, since chromosome size was only associated with the FOM orfs model, it is possible that the chromosome size confounds with AT content, or one of the other factors, and is thus found significant by the regression models. Habitat was found to improve the coefficient of determination (*R^2^*) slightly but only for the ZOM and SOM orf regression models. It is therefore possible that habitat is confounding with another covariate, just as in the case for chromosome size. Most variance was explained by the FOM and FOM orfs regression models (*R^2^ = 0.83*), while the least variance was explained by the SOM orfs model (*R^2^ = 0.58*). The orfs models were in general better, in terms of variance explained (Table 3), than the models based on whole chromosomes, and, from Figure 5, it can be seen that they in general obtained higher PCH scores.

**Table 3:** PCH regression AIC/Coefficient of variation scores.

	Constant	Size	AT	OUV	Phyla	Oxygen	Habitat	Transform
ZOM	−728		−1051, R^2^ = 0.37	−1492, R^2^ = 0.67	−1727, R^2^ = 0.77		−1730, R^2^ = 0.77	λ = 10
ZOM orfs	−827		−1240, R^2^ = 0.45	−1629, R^2^ = 0.68	−1740, R^2^ = 0.74	−1753, R^2^ = 0.74		λ = 17
FOM	−828		−1364, R^2^ = 0.4	−1831, R^2^ = 0.8	−1894, R^2^ = 0.82	−1920 R^2^ = 0.83		λ = 4
FOM orfs	−715	−816, R^2^ = 0.14	−1361, R^2^ = 0.61	−1847, R^2^ = 0.8	−1902, R^2^ = 0.82	−1924, R^2^ = 0.83		λ = 9
SOM	−1088		−1278, R^2^ = 0.24	−1845, R^2^ = 0.66	−2032, R^2^ = 0.75	−2051, R^2^ = 0.76		λ = 3
SOM orfs	−1059		−1306, R^2^ = 0.3	−1460, R^2^ = 0.44	−1636, R^2^ = 0.58	−1665, R^2^ = 0.59	−1666, R^2^ = 0.6	λ = 3

Results of forward fitting regression models with the response variable in the leftmost column followed by the predictors used in the models.

The ZOM PCH compared to FOM PCH scores obtained a coefficient of determination score of *R^2^ = 0.38*, while ZOM PCH compared to SOM PCH scores were found to have a *R^2^ = 0.21*. Similar to the FOM and SOM OUV scores, the FOM compared to SOM PCH scores obtained the highest coefficient of determination with *R^2^ = 0.52*. Hence, corresponding to the results obtained for the OUV values, ZOM PCH was more similar to FOM PCH than SOM PCH.

Both OUV and PCH based regression models were also tested with pathogenicity as a factor. This factor is assumed to give a weak indication of recombination or horizontal transfer [30], [31], but was not found significant for any of the models and therefore removed.

## Discussion

### OUV-Based Models and their Association with Genomic Signatures

The Markov model based genomic signatures discussed here differentiate organisms in terms of the ratio of genomic tetranucleotide frequencies divided by Markov chain approximated tetranucleotide frequencies. OUV values, or the variance between genomic tetranucleotide frequencies and approximated tetranucleotide frequencies, are therefore strongly associated with genomic signatures, since the bias in tetranucleotide usage drives the genomic signature in the respective organism. Factors affecting Markov model approximated OUV values in prokaryotes were examined using regression analysis. The regression models revealed that OUV is more associated with AT content than phyla. The relationship between OUV and AT/GC content is most likely also confounded with factors not specified in the model, since genomic AT content has been associated with environment [11], [12]. Habitat, a categorical factor describing the environment where the organisms are usually found, was divided into five branches: aquatic, host-associated, terrestrial, specialized (extremophiles) and multiple (same species found in many different environments). The regression models, except FOM OUV, improved with the inclusion of the habitat factor for all measures. It is assumed that the lack of significant association between the FOM OUV measure and habitat is due to the coarseness of the methods used. The same can be said for the categorical variable specifying oxygen requirement. The oxygen requirement variable describes aerobic, anaerobic and facultative lifestyles, and was found to be significantly improving all regression models except for the ZOM OUV model.

The coefficient of determination (*R^2^*) is in general higher for all OUV models restricted to open reading frames, indicating that the variances in the regression models are better explained in the coding regions. The oligonucleotide based genomic signature methods require relatively large segments of DNA to give meaningful results, *i.e.* at least multiple kbp's depending on the Markov model used [25]. The non-coding regions were therefore not separated from the chromosomes analyzed. Hence, difference between coding and non-coding regions was measured as the difference between chromosomes, containing both coding and non-coding regions, and predicted open reading frames. It is interesting to note that AT content explains more variance in the OUV models than phyla. An explanation may be that the genomic DNA composition of prokaryotes is more sensitive to changes in conditions affecting mononucleotide frequencies than phyla. In other words, phyla could provide prokaryotic genomes with a sense of ‘inertia’ (or memory) while environmental factors affecting base composition may be responsible for inducing more rapid genomic changes. For instance, nitrogen is more abundant in GC rich genomes meaning that changes in nitrogen levels may affect the base composition in such genomes severely [32]. Similar trends have been observed for oxygen and aerobic bacteria, in the sense that the genomes of aerobic bacteria tend to be more GC rich [33]. In general, it has been shown, using sequenced genomes, that the environment affects the base composition in bacteria [11], and that the resulting change is relatively fast [12].

GC rich genomes were found to be more strongly biased in terms of OUV than AT rich genomes in the sense that AT rich genomes had, on average, a more random walk like DNA composition. Lower OUV scores mean less bias which, in turn, implies increased independence between the adjacent nucleotides and therefore more random genomic sequence patterns, presumably due to increased mutation rates [14]. This is supported by the observation that intracellular bacteria having undergone genome reduction tend to lose DNA repair genes and become AT rich [34]–[36]. This appears to happen to free living genomes as well when the amount of available nutrition changes. An example of the latter can be found in different strains of the ocean living bacterium *Prochlorococcus marinus*. Some of the *P. marinus* strains that live in the upper high light layer of the ocean tend to have smaller genomes than strains living in the nutrition rich low light areas [37]. Although only slightly, AT content was associated with habitat for host associated and terrestrial environments (*p<0.001*), but aquatic, multiple (bacteria found in different environments) and specialized habitats (extremophiles) were not found significant. Oxygen requirement was also associated with AT content, but only slightly for anaerobic and facultative oxygen requirement (*p<0.001*). In contrast, growth temperature was not significantly (*p>0.5*) associated with AT content. It should be emphasized that global genomic data is necessarily “noisy”, and many of the environmental influences are assumed to affect particular areas of the genome and in distinct patterns [38]. Examinations of environmental influences on more specific genomic regions will, however, require the use of different methods than those employed here. It is conceivable that such methods should be based on nucleotides rather than oligonucleotides for an increase in sensitivity [39], [40].

The SOM OUV method has also been used to approximate oligonucleotide frequencies in *E. coli* [41], [42]. The SOM method was found to be inferior to similar methods allowing gaps [42]. Our findings indicate that the quality of the oligonucleotide approximations in prokaryotes depend, most importantly, on AT content. Thus, since AT rich genomes tended to be less biased, in terms of random walk like sequence patterns, than GC rich genomes, it may indicate that AT rich genomes are more concentrated, that is, dependencies between nucleotides are more short ranged, and therefore easier to approximate.

### Variance of Genomic Signatures within Genomes

**Figure 6:**
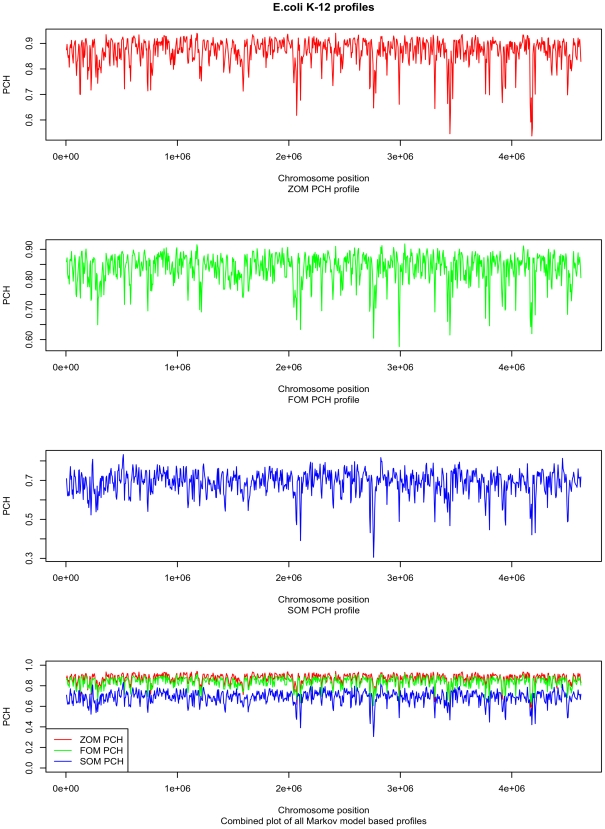
*E. coli K-12* profiles based on ZOM, FOM and SOM PCH measures. Plots of genomic signatures based on ZOM (red line), FOM (green line), or SOM (blue line) models compared with tetranucleotide-based signatures from a 10 kbp sliding window, overlapping every 5 kbp. Higher PCH (vertical axis) mean greater intra-chromosomal homogeneity. The low dips located close to genomic positions (horizontal axis) 2.1 mbp and 2.8 mbp indicate prophage DNA.

The principal motivation for this work was to examine prokaryotic genome homogeneity using Markov chain based genomic signatures. Figure 6 shows how the genomic signature changes within an *E. coli K-12* genome. The ZOM PCH measure obtained higher scores than the FOM PCH measure, which, in turn, obtained higher scores than the SOM PCH measure. It can be seen that PCH scores increase with wider sliding windows [25].

The regression models indicate that all PCH methods are influenced by AT content and phyla, but most of all, corresponding Markov chain model OUV scores. Thus, genomic homogeneity, as measured using Markov chain based genomic signatures, is positively correlated with bias in genomic tetranucleotide patterns in the sense that the less random walk like the DNA composition of a genome is, the more homogeneous the genome is.

The FOM PCH based regression model obtained a coefficient of determination higher than the other Markov-chain based PCH models. In other words, FOM PCH was the best regression model in terms of variance explained. Although the reason for this is not known, it has been shown that mono- and dinucleotide frequencies to a large degree determine genome wide codon usage bias, and that the codon bias can be determined from intergenic regions as well [43]. Codon bias is therefore found to be, first and foremost, determined by forces inducing mutations on the whole genome and only secondary by factors related to specific genes [43]. The SOM PCH based models obtained *R^2^* values lower than those of both ZOM and FOM PCH models. The low PCH scores obtained with the SOM-based measures may indicate that the lower *R^2^* values obtained with the SOM PCH regression models may be caused by the increased genetic ‘noise’ found in these models.

The correlation between OUV and PCH scores means that random walk like DNA composition is strongly associated with intra-genomic heterogeneity, as measured by the different Markov model based genomic signatures. All PCH models, except for the ZOM PCH model, improved significantly with the inclusion of the oxygen requirement factor, although only slightly in terms of AIC and *R^2^*. This result may indicate that oxygen requirement affects DNA composition in prokaryotes on many levels. Oxygen requirement did not reach the same significance level in the ZOM PCH model (*p = 0.08*) as the other models.

A small, but significant, improvement to the ZOM and SOM PCH orfs models was observed with the inclusion of the habitat factor. Chromosome size was only found to improve the FOM PCH orfs model. These results mean that chromosomal homogeneity, in terms of variance in the Markov model based genomic signatures, is associated with, first and foremost, corresponding ZOM, FOM and SOM OUV scores followed by AT content and phyla, with oxygen requirement influencing chromosomal homogeneity to a lesser degree.

Although all Markov-chain based PCH measures, and particularly the SOM PCH model, are fairly crude in measuring average chromosomal homogeneity it was of some surprise to note the substantial improvement to the models by the inclusion of AT content as a factor. All statistical models improved considerably in terms of both AIC and *R^2^* scores. This was unexpected since the variance of genomic signatures within genomes has usually been associated with foreign genetic elements like phages and pathogenicity islands [21]. The finding that global AT content is an important factor associated with how the genomic signatures vary within genomes can be seen from tables 4–9, where the high PCH scoring genomes tend to have lower AT content than the low PCH scoring genomes. The strong association with the corresponding OUV values may be a consequence of selective forces. Indeed, AT content is associated with phyla in the sense that similar species and strains tend to have similar AT content. However, all statistical PCH models indicated that AT content contributed more to the regression models than phyla. It should be noted that the above mentioned results are trends with a varying proportion of unexplained variance, *i.e.* exceptions do occur. In addition, the selection of sequenced genomes is in turn biased both by genome size and interest.

**Table 4:** Highest ZOM PCH scoring genera.

Name	NCBI accession number	ZOM PCH	AT	Size mbp	ZOM OUV/Z-scores (log)	Oxygen	Habitat	Growth temperaure
Candidatus Korarchaeum cryptofilum strain OPF8	NC 010482	0.96	0.51	1.59	4.32E-006/0.96	Anaerobic	Specialized	Thermophilic
*Rhodopirellula baltica* SH 1	NC 005027	0.96	0.45	7.15	3.59E-006/0.63	Aerobic	Aquatic	Mesophilic
*Wolinella succinogenes*	NC 005090	0.96	0.52	2.11	4.61E-006/1.07	Aerobic	Host associated	Mesophilic
*Dichelobacter nodosus* strain VCS1703A	NC 009446	0.96	0.56	1.39	6.26E-006/1.7	Anaerobic	Host associated	Mesophilic
*Leptospira borgpetersenii* serovar Hardjo-bovis strain JB197	NC 008510	0.96	0.6	3.58	5.08E-006/1.25	Aerobic	Host associated	Mesophilic

**Table 5 pone-0008113-t005:** Lowest ZOM PCH scoring genera.

Name	NCBI accession number	ZOM PCH	AT	Size mbp	ZOM OUV/Z-scores (log)	Oxygen	Habitat	Growth temperaure
*Buchnera aphidicola*	NC 004545	0.73	0.75	0.62	1.99E-006/-0.41	Facultative	Host associated	Mesophilic
*Staphylococcus epidermidis* strain RP62A	NC 002976	0.73	0.68	2.62	7.62E-007/-2.11	Facultative	Host associated	Mesophilic
Candidatus Blochmannia floridanus	NC 005061	0.75	0.73	0.71	9.18E-007/-1.78	Aerobic	Specialized	Mesophilic
*Bacillus cereus strain* ATCC 14579	NC 004722	0.77	0.65	5.22	1.24E-006/-1.31	Facultative	Multiple	Mesophilic
*Finegoldia magna* strain ATCC 29328	NC 010376	0.78	0.68	1.8	2.47E-006/-0.03	Anaerobic	Multiple	Mesophilic

**Table 6 pone-0008113-t006:** Highest FOM PCH scoring genera.

Name	NCBI accession number	FOM PCH	AT	Size mbp	FOM OUV/Z score (log)	Oxygen	Habitat	Growth temperaure
*Caldivirga maquilingensis* strain IC-167	NC 009954	0.92	0.57	2.08	2.36E-006/1.95	Aerobic	Specialized	Thermophilic
*Helicobacter acinonychis* strain Sheeba	NC 008229	0.92	0.62	1.55	1.28E-006/0.87	Aerobic	Host associated	Mesophilic
*Dehalococcoides strain* CBDB1	NC 007356	0.91	0.53	1.4	1.36E-006/0.98	Anerobic	Multiple	Mesophilic
*Pyrobaculum aerophilum*	NC 003364	0.91	0.49	2.22	1.32E-006/0.93	Facultative	Aquatic	Thermophilic
*Ignicoccus hospitalis* strain KIN4 I	NC 009976	0.91	0.43	1.36	1.5E-006/1.15	Anaerobic	Aquatic	Thermophilic

**Table 7 pone-0008113-t007:** Lowest FOM PCH scoring genera.

Name	NCBI accession number	FOM PCH	AT	Size mbp	FOM OUV/Z score (log)	Oxygen	Habitat	Growth temperaure
*Fusobacterium nucleatum*	NC 003454	0.46	0.73	2.17	8.88E-007/0.23	Anaerobic	Host associated	Mesophilic
*Mycoplasma penetrans*	NC 004432	0.46	0.74	1.36	3.87E-007/-1.23	Facultative	Host associated	Mesophilic
*Borrelia afzelii* strain PKo	NC 008277	0.53	0.72	0.91	3.69E-007/-1.31	Aerobic	Host associated	Mesophilic
*Parachlamydia* sp. strain UWE25	NC 005861	0.55	0.65	2.41	8.98E-008/-3.05	Aerobic	Host associated	Mesophilic
*Clostridium difficile* strain 630	NC 009089	0.56	0.71	4.29	6.34E-007/-0.36	Anaerobic	Multiple	Mesophilic

**Table 8 pone-0008113-t008:** Highest SOM PCH scoring genera.

Name	NCBI accession number	SOM PCH	AT	Size mbp	SOM OUV/Z score (log)	Oxygen	Habitat	Growth temperaure
*Helicobacter acinonychis* strain Sheeba	NC 008229	0.87	0.62	1.55	4.12E-007/1.31	Aerobic	Host associated	Mesophilic
*Thermoproteus neutrophilus* strain V24Sta	NC 010525	0.86	0.4	1.77	8.74E-007/2.88	Anaerobic	Specialized	Thermophilic
*Ignicoccus hospitalis* strain KIN4 I	NC 009776	0.86	0.43	1.3	4.79E-007/1.63	Anaerobic	Aquatic	Thermophilic
*Methanococcus aeolicus* strain Nankai-3	NC 009635	0.84	0.7	1.57	3.22E-007/0.8	Anaerobic	Aquatic	Mesophilic
*Methanoculleus marisnigri* strain JR1	NC 009051	0.84	0.38	2.48	5.3E-006/1.84	Anaerobic	Aquatic	Mesophilic

**Table 9:** Lowest SOM PCH scoring genera.

Name	NCBI accession number	SOM PCH	AT	Size mbp	SOM OUV/Z score	Oxygen	Habitat	Growth temperaure
*Kineococcus radiotolerans* strain SRS30216	NC 009664	0.27	0.26	4.76	7.84E-007/2.65	Aerobic	Multiple	Mesophilic
*Mycoplasma penetrans*	NC 004432	0.4	0.74	1.36	1.95E-007/-0.24	Facultative	Host associated	Mesophilic
*Ehrlichia ruminantium* strain Gardel	NC 006831	0.4	0.72	1.5	1.7E-007/-0.53	Aerobic	Host associated	Mesophilic
*Nocardioides* sp. strain JS614	NC 008699	0.43	0.28	4.99	4.85E-007/1.65	Aerobic	Multiple	Mesophilic
*Fusobacterium nucleatum*	NC 003454	0.44	0.73	2.17	2.81E-007/0.52	Anaerobic	Host associated	Mesophilic

### Genomic OUV and PCH Scores as Measures of Selection Forces

It is reasonable to think that OUV mirrors, although somewhat crudely, the sum of selective forces acting on an organism's genomic DNA. Low OUV scores implies that the observed genomic DNA composition is closer to a model assuming, in the simplest case (ZOM), only similar mononucleotide frequencies. Thus, the more similar the genomic DNA composition, measured as mononucleotide frequency approximated tetranucleotide frequencies, is to corresponding mononucleotide frequencies, the weaker selective forces are assumed to have been acting on the genome. It has also been noted in several articles [34], [36], that genomes in a stable environment, such as in a nutrition providing cell, tend to lose DNA repair genes with the implication that genomes mutate, particularly from cytosine to thymine on the lagging strand [44], leading subsequently to many defective genes and, ultimately, reduced genomes [34]. To reverse the processes of genome reduction, stronger selection forces must act on the genome. There are not many examples of genome expansion known to the authors, however *Ehrlichia ruminantium* and *Frankia sp.* strain EAN1pec are assumed to be affected by stronger selection forces due to their alleged genome increase [45], [46]. The strong association between OUV and PCH scores may indicate that strong selection forces, *i.e.* high OUV and PCH scores, have a high impact on an organisms DNA sequence which results in higher chromosomal homogeneity. This may explain the association between AT content and OUV/PCH scores, which, furthermore, may imply that genomic amelioration rates [47] are linked to AT content.

In summary, homogeneity in prokaryotic genomes, measured using genomic signatures, is highly associated, in order of importance, with bias in DNA composition, as measured by the OUV measure, AT content, phyla and oxygen requirement. All Markov-chain based genomic signatures were found to be associated with AT/GC content, with the implication that the more GC rich and higher OUV a genome has, the more homogeneous is the genome. In other words, GC rich genomes tend to be more homogeneous than AT rich. This result was not expected since genomic signatures are known to be sensitive to foreign genetic elements. Other factors such as habitat and oxygen requirement were also significant factors for the different models, and the genomic signatures were more stable in coding regions than in non-coding regions.

## Materials and Methods

All 636 genomes, consisting of 694 prokaryotic chromosomes, were downloaded from the NCBI database [48] [http://www.ncbi.nlm.nih.gov/genomes/lproks.cgi]. Genomic properties and information about the different organisms were also obtained from the NCBI website [48]. Regression analyses and data visualization was performed with R [49], and computer programs were made according to the guidelines described below. DNA sequences were analyzed in the 5′ → 3′ direction. All data used in the analyses, can be found as supporting information (File S1).

### Notation

Using the notation from Karlin and co-workers [20], the ZOM, FOM and SOM based functions are represented by the following formulas:
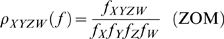


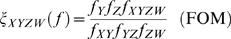


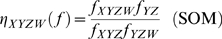

*f* is the DNA sequence while *f_XYZW_* indicates the frequency of oligo *XYZW* in *f*. *f_X_*, *f_XY_*, and *f_XYZ_* represents mono- to trinucleotidefrequencies of *X*, *XY* and *XYZ* in DNA sequence *f*, respectively.

The Pearson correlation formula was used to compare different DNA sequences *f* and *g*:
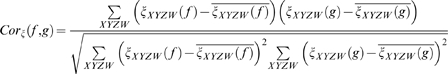



This comparison was carried out using the FOM model, and the sums are taken over every possible tetranucleotide combination XYZW.

To measure how the genomic signature changed within the different genomes, an average correlation score was calculated based on the ZOM, FOM and SOM measures above together with the correlation formula. Thus, the variance of the different ZOM, FOM and SOM-based genomic signatures were examined within each chromosome by comparing whole-chromosome signatures to signatures obtained from a non-overlapping sliding window of 20 kbps using the Pearson correlation formula. The average value for each chromosome was in turn calculated from the correlation scores between each sliding window and the whole chromosome signature.

The maximum number of sliding windows *S* is given by:
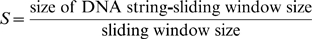



The ZOM, FOM and SOM based OUV measures calculate the variance between observed and approximated oligonucleotide frequencies:
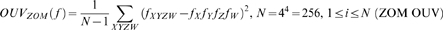


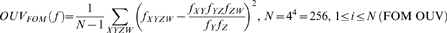


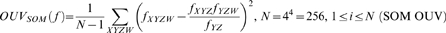



### Regression Analysis

The models measuring associations between OUV values as response functions and chromosome size, AT content, phyla, habitat, oxygen requirement and growth temperature as predictors, were all based on transformed ‘linear’ regression analysis:
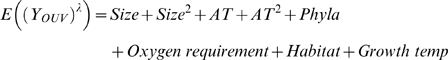



All PCH models were on a similar form, but with OUV included as a factor:
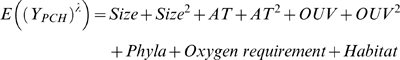



All regression equations explained in this work were transformed on the left hand side with the *λ* coefficient found using Box-Cox estimation [50] to conform as much as possible to the underlying hypothesis of normally distributed residuals. Phyla, oxygen requirement, habitat and growth temperature were all categorical variables, while PCH, Size, AT and OUV were numerical variables.

The results obtained must be considered as coarse as there is some expected co-linearity between predictors like OUV, AT content and chromosome size [14], [15], [29], [51]. In addition, the computed oligonucleotide frequencies were all obtained by counting overlapping oligonucleotides, thereby adding considerable ‘noise’ to any potential genomic signal. The quality of the models was assessed using the Akaike information criterion (AIC) and the coefficient of determination (*R^2^*). Factors were added forwardly to the models and deleted if *p>0.001*. The Z-scores, i.e. *(Z-μ)/σ*, in tables 4–9 are based on transformed OUV values.

## Supporting Information

File S1 Main dataset. An Excel file containing the data used to generate the results in the paper
(0.26 MB XLS)
